# Testing the Mystic School Mobile Application to Promote Active Commuting to School in Spanish Adolescents: The PACO Study

**DOI:** 10.3390/children9121997

**Published:** 2022-12-19

**Authors:** Romina Gisele Saucedo-Araujo, Francisco Javier Huertas-Delgado, Yaira María Barranco-Ruiz, Isaac José Pérez-López, Susana Aznar-Laín, Palma Chillón, Manuel Herrador-Colmenero

**Affiliations:** 1 Department of Physical Education and Sports, PROFITH “PROmoting FITness and Health through Physical Activity” Research Group, Sport and Health University Research Institute (iMUDS), Faculty of Sport Sciences, University of Granada, 18011 Granada, Spain; 2 La Inmaculada Teacher Training Centre, University of Granada, 18013 Granada, Spain; 3 Department of Physical Education and Sports, PROFITH “PROmoting FITness and Health through Physical Activity” Research Group, Sport and Health University Research Institute (iMUDS), Faculty of Education and Sport Sciences, University of Granada, 52071 Melilla, Spain; 4 Department of Physical Education and Sports, “Educación Física y Transformación Social”, SEJ546 Research Group, Faculty of Sport Sciences, University of Granada, 18071 Granada, Spain; 5 PAFS Research Group, Faculty of Sports Sciences, University of Castilla-La Mancha, 13071 Toledo, Spain; 6 CIBER of Frailty and Healthy Aging (CIBERFES), 28029 Madrid, Spain

**Keywords:** exercise, health, technologies, physical education lesson, school-based intervention

## Abstract

Active commuting to and/or from school (ACS) is an opportunity to increase daily physical activity (PA) levels in young people. Mobile-device interventions focused on promoting the practice of health-related PA can be more cost-effective than traditional interventions in this population. Objective: To analyze the adolescents’ opinion of the mobile application (app) Mystic School, which was designed to promote ACS in Spanish adolescents. Methods: A total of 44 students (14–15 years old) from Granada and Jaén participated in the test of the Mystic School app during two phases: phase 1 (*n* = 10) for 2 weeks and phase 2 (*n* = 34) for 1 month. Each phase included an app presentation, a follow-up, and focus group sessions. The qualitative analysis was carried out through NVivo software. Results: In phase 1, adolescents reported improvements in the design and functioning, such as the avatar movement, virtual steps utilities, and multiplayer function. These suggestions were included in phase 2. After phase 2, adolescents reported that it is important to add the possibility of playing without an Internet connection to the game, to include more competitive options, prizes, and to increase the difficulty of the levels. In both phases, problems with the step number counting remained. Conclusion: The Mystic School app can be a useful tool for the physical education teacher to integrate the content from this curriculum related to the promotion of PA, such as ACS.

## 1. Introduction

The socio-ecological model for active living [1] comprises four domains of physical activity (PA): recreation activities, active transport, occupational activities, and household activities. Consequently, increasing the time devoted to the active-transport domain throughout the day is important to achieve an active lifestyle. In this sense, active commuting to and/or from school (ACS) should be important for young people. ACS is defined as the use of active modes of transportation, such as walking, cycling, or skateboarding, which employs energy expenditure for commuting to and/or from school [2]. Thus, ACS has been considered an alternative for improving individual health by accumulating daily PA [3], as well as providing many benefits, such as improvements to body composition, cardiorespiratory fitness [4,5], and psychological health with better self-efficacy and autonomy [6]. Additionally, ACS offers benefits for the environment, such as reductions in air pollution and traffic jams [7]. Despite the individual and social benefits, the trends in ACS have decreased in some countries (i.e., the Czech Republic [8], the United States [9], England [10], and Canada [11]). A recent Spanish study [12] showed that around 60% of adolescents used ACS, remaining stable between 2010 and 2017. According to the scientific literature on the interventions that were carried out, there is a need for high-quality and attractive interventions to maintain or increase ACS behavior in children and adolescents [2,13,14].

From this perspective, the growing number of mobile-device users has created opportunities to develop attractive mobile applications (apps) [15] that convert the mobile phone into an effective tool to increase adherence to PA interventions [16]. Accordingly, mobile apps are attractive to young people because of their easy daily use and the large number of options. In this line, an alternative strategy to replace passive with active screen time is using the mobile device through active video games (AVGs), which require PA to play the game, more than conventional hand-controlled games [17]. In the last few years, AVGs, or “exergames”, emerged as an innovative intervention to increase PA levels with the aim of reducing childhood obesity [18].

AVG-based interventions to increase PA levels have been frequently implemented from primary schools to universities [19,20,21]. In concordance with a recent systematic review, AVGs can be an effective tool for adolescents [22]. Therefore, due to their potential benefits, AVGs need to be investigated in depth. Qualitative research through focus groups is currently used as a self-contained method and in combination with other research methods, such as surveys or in-depth interviews [23]. Focus groups are very useful in testing new products or concepts in order to guarantee their success [24], as is the case in the present study. Consequently, to ensure the success of the app, it is necessary to know the users’ opinions.

To our knowledge, even though ACS is a potential PA domain to be promoted in young people, there are few interventions or tools based on AVGs using mobile apps that have been developed and tested considering the opinions of adolescents. Thus, the aim of this study was to analyze the adolescent’s opinion of the mobile app Mystic School, designed to promote ACS in Spanish adolescents.

## 2. Materials and Methods

### 2.1. Study Design 

This study adopted a qualitative research methodology based on focus groups. This type of qualitative methodology consists of a group of individuals selected and assembled by researchers to discuss and comment on, from their personal experience, the subject of the research [24]. Specifically, in the present study, an AVG app called Mystic School was the product analyzed and improved by focus groups considering their user experience. 

### 2.2. Description of the Mystic School App

**Figure 1:**
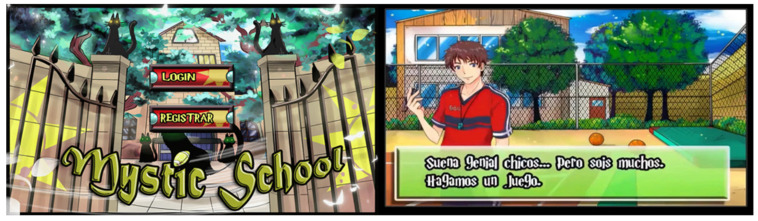
Registration in the video game and first explanation screen (Spanish version).

The Mystic School is a mobile app based on an AVG whose main purpose is to encourage walking as a mode of commuting to increase the levels of daily PA in young people. The Mystic School software was designed for the Android Operation System from the Spanish 4.0 version (Ice Cream Sandwich) and posteriors. For its functionality, the Mystic School app includes an accelerometer and a GPS to record the number of steps and distance. Real steps while adolescents walk during the day are transformed into virtual steps to complete the game. These virtual steps allow players to move an avatar through the different Mystic School screens, which are organized as different levels inside a maze. The video game is placed in a school context. The first screen (level 1) starts when the player finishes a physical education (PE) lesson, and the teacher asks the player and his/her classmates to help pick the material up. From here, the players need to accumulate real steps to be able to move the avatar within the game in order to collect the materials that the PE teacher has requested. In the storage room, they find a strange ball that transports them to a school in ruins, located in the parallel universe of Mystic School (Figure 1). Every task completed in the game allows them to obtain rewards to continue advancing to the different levels of the game through different screens.

**Figure 2:**
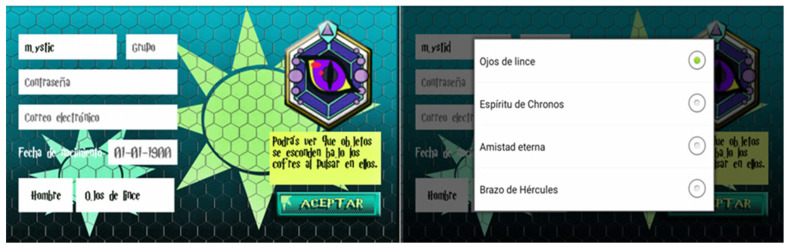
User registration and special skill selection (Spanish version).

The Mystic School app may be played individually or with up to 3 players (in the same group). Each adolescent must choose an avatar. The virtual steps may help the students obtain different objects and prizes while they advance toward the last level. In addition, in the app, the student can choose a “special skill” (see Figure 2). Each skill is an advantage for each player (two players cannot have the same skill in the same group). 

The four skills are:

- “Eyes of Lynx”: Discover the contents of the treasure chest (in each level of play, there are different chests with hidden objects) before it is opened;
- “Spirit of Chronos”: Recorded steps are worth twice as much when collected during a 30 min period per day (e.g., from 12:00 to 12:30);
- “Eternal Friendship”: Share steps with another avatar in the same group;
- “Arm of Hercules”: Share an item with another avatar in the same group.

### 2.3. Participants and Recruitment 

Participants of this qualitative study belonged to the “Pedalea y Anda al cOle: PACO” (Cycling and Walk to School) study. The PACO study was designed to encourage ACS among Spanish students within the PE curriculum for compulsory secondary education (14–15 years old) [25]. The PACO study was approved by the Review Committee for Research Involving Human Subjects at the University of Granada (Reference: 162/CEIH/2016). For this qualitative study, participants were recruited in two different phases (phase 1 and phase 2) from four public schools in the cities of Granada, Jaén, and Toledo. In phase 1, the sample was selected via convenience from Granada (non-randomized sampling) in the 2016–2017 academic year, whereas, in phase 2, a random sampling from Granada, Jaén, and Toledo was used (the 2018–2019 academic year). Initial contact was made with the PE teacher and the school staff at secondary schools to explain the study to them. Then, according to the selection criteria of the study explained below, for the secondary schools that agreed to participate, we sent an informed-consent form to the students’ legally authorized representatives (parents/legal guardians). Afterward, the physical education teacher collected the parents’ informed consent. 

The following inclusion criteria were applied to the participants: (1) attend 3rd-grade compulsory secondary education (14–15 years old), (2) have an Android Operation System mobile phone, (3) have access to the Internet, and (4) play the Mystic School app during the intervention. 

### 2.4. Procedure 

#### 2.4.1. Phase 1

Adolescents from 2 high schools located in Granada (Spain) were invited to take part in the study. A total sample of 14 participants (6 boys and 8 girls) played the Mystic School app during the testing period (two weeks). Phase 1 included one app presentation session, two weeks to test the Mystic School app, and, finally, one focus group session (see Table 1).

The contents of “Session I. Mystic School presentation” were (1) an introductory presentation of the Mystic School app; (2) a brief tutorial of how to download the Mystic School app; (3) a demonstration of the steps to install the application; and (4) once the Mystic School app was installed, the research team explained the characteristics of the game. 

The PE teachers at both schools supported us all the time to encourage the students to participate. However, not all of the adolescents could participate because they had limited access to the Internet on their mobile phones. Another reason was that some adolescents were in an exam period or they were not able to use their mobile phones during the week. 

The contents of “Session II. Focus group” were based on carrying out a focus group implemented two weeks after experiencing and playing the Mystic School game. Only the participants that played during the two weeks were invited to join the focus group (during the sessions, it was recorded who had attended and played during the indicated period). Four participants did not attend a focus group because one had a broken phone and the others had no time to play. The same researcher who led the meetings with the students and the full process acted as the moderator (who guided the group). The focus group sessions had an average duration of 15 min. The topics were discussed in the groups anonymously. During the focus group sessions, a total of 20 questions (File S1 (Supplementary Materials)) were discussed. After analyzing the registers of the focus group, the questions were divided into seven categories: (1) the usability of the app; (2) the assessment of the design; (3) the usefulness of social networks in the game; (4) an understanding of the degree of usefulness in the game; (5) an understanding of the game and the design; (6) the usability of social networks in the game; and (7) the overall user satisfaction and impact on daily habits. These contributions made by the participants were discussed by the research team to be incorporated into the app.

#### 2.4.2. Phase 2

The Mystic School app was implemented over 4 sessions in 1 month (1 session per week) during the PE lessons (see Table 2).

**Figure 3:**
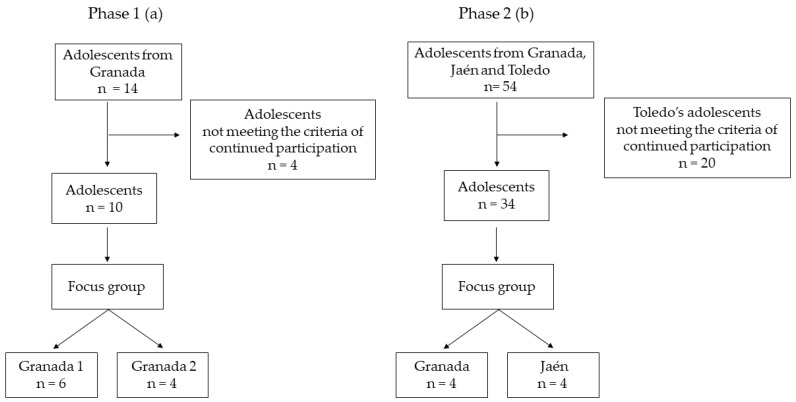
Flowchart of participants for phase 1 (plot **a**) and phase 2 (plot **b**).

After the 4 sessions, the participants from the class groups located in Toledo (*n* = 20) did not meet one inclusion criterion (use the app for one month), and they were excluded. Therefore, they were not invited to take part in the focus group. A total of 34 students participated in the study. A focus group of 4 participants was held in Granada, and a second focus group of 4 participants was held in Jaén. They participated in the entire session, and they talked about their user experiences (Figure 3).

Focus group: The mean duration of the focus groups was approximately 10 min. As a reward for their participation, the focus group participants received a healthy breakfast. 

### 2.5. Data Analysis

The text of both focus groups was analyzed through qualitative methods using the software NVivo 11 plus. The following phases were carried out:

(1) Transcriptions were read several times to obtain a sense of the overall data;
(2) The text was divided into meaning units;
(3) The meaning units were coded, and these codes were compared, contrasted, and sorted into themes, while maintaining fidelity with the text.

## 3. Results

The results obtained from the focus group questions were organized in two parts: (1) the adolescents’ perception of the app and (2) aspects to improve in terms of design, operation, and general satisfaction. The results will be organized in the two phases previously indicated in the study.

Phase 1

The comments in both groups were: (a) the game did not correctly record the steps, or there were more steps without having moved from the site; (b) sometimes, it did not work; and (c) they wanted more personalization of the avatar. Many comments were consequences of the situation that the game did not correctly record the steps, which was a main technical issue (Table 3).

Although the degree of satisfaction was positive, the students indicated that they would return to play if the avatar’s movement in the maze improved. Therefore, they found several software bugs that made the daily use of the app difficult. The most relevant comments were that the Mystic School app did not work correctly and it did not count the steps as the player expected. On the other hand, the students suggested improvements, such as changing the avatar (being customizable), more competitive tasks, and being able to play single and multiplayer. The adolescents played an average of 4 out of 10 levels. Because of the previous reasons, the adolescents provided a low use of the app and, consequently, few opinions. 

The computer developers focused on correcting app errors, such as not recording the steps correctly, leaving the blank screen unexpectedly, and technical issues (i.e., the brightness of the animation). In addition, the computer experts improved the functioning of the GPS. Therefore, the avatar’s way of movement was changed, as mentioned by the students prior to phase 2. 

Phase 2

The participants highlighted aspects for improvement in terms of the design of the Mystic School app, the time they spent playing, and what they liked most about the app (Table 4).

In relation to the content “suggestions for improvement”, students answered the first question: “What aspects would improve the Mystic School app?” They stressed the use of the mobile app without an Internet connection since some of them did not have a service that provided Internet. They also commented that more prizes should be provided during the game as rewards for reaching a certain number of steps. In addition, they proposed an increment in competitiveness and difficulty during the different levels of the game.

In the content “playing time” (second question), “How much time have you spent playing?”, it was observed that some of the participants had not played enough. There were different reasons: (a) the software did not work; (b) they had many extracurricular activities; and (c) they had many exams. Finally, the participants were asked what they liked most about the game. About the content’s “positive aspects”, the participants said that the app was an original idea, and the design was good. 

## 4. Discussion

In the current study, a mobile app was developed to promote ACS in adolescents. The participants found the Mystic School app a fun alternative for play, despite some technical problems, such as when recording steps. In addition, the adolescents suggested some improvements to make the application work better.

A meta-analysis [16] provided evidence that the effectiveness of mobile phone apps in increasing PA is better in the short term. Furthermore, the reason can be the intensity of the player activity, due to adolescents often losing interest in playing games for longer periods. Randomized controlled trial designs, larger sample sizes, and validated activity measurements beyond the school day are needed. Limited evidence is available on the long-term efficacy of AVGs for PA promotion [26].

The perception of the Mystic School app was positive. The students liked playing in groups because they could share experiences. Once again, the need for socialization and cooperative learning among all members is confirmed [27]. Thus, identifying what makes an app fun and engaging is important for an optimal game design [28]. It is important to focus on more user-identifiable characters, such as high-level realistic graphics and well-defined instructions; this has been suggested by a recent systematic review as one of the most important points for a successful healthy-lifestyle promotion [29]. In addition, the adolescents wanted more competitive challenges. Consequently, initial gamification mechanisms, such as competitions and challenges, were used and increased during the intervention because competitiveness was found to be associated with greater enjoyment [30]. In addition, Shameli et al. [31] observed that during walking competitions, the average user increases PA by 23%. 

According to Sallis et al. [32], who recommended that interventions should provide greater incentives, the final focus group was given the reward of a healthy breakfast. In addition, each adolescent received a bracelet and a backpack (by raffle) for their participation. Moreover, points-based reward systems were implemented to increase student commitment, which only worked at first. As previous literature confirmed [33], this type of system seems not to have a long-term impact. However, making something enjoyable depends on intrinsic motivation based on satisfying fundamental needs (i.e., relatedness, competence and autonomy [34]), but current AVGs have failed to adequately meet all of these needs. In our study on AVGs, the researchers should have provided more motivational tools for the adolescents to engage them in play and keeping their adherence to accumulating steps. 

In light of the results, it is important to use different resources to increase PA in adolescents, and smartphone apps are crucial in this process. Around 94% of adolescents own or have access to smartphones, and 89% of them indicate that they access the Internet almost constantly or several times a day [35]. In addition, a number of different mobile apps are now available that increase PA, such as Pokémon Go [36] and Zombie Run [37]. The evidence of a meta-analysis showed that a smartphone-based intervention might be a promising strategy to increase steps in young people [38]. Therefore, these tools can have different purposes if the right approach is designed to motivate adolescents to change their behaviors, such as increasing PA. After the implementation of this AVG, the adolescents showed interest and initiative to use it. This type of proposal can be a useful tool to complement or add to the PE curriculum, although it should be analyzed to corroborate if it increases PA. Another lesson learned from the implementation of the AVG is that the difficult technical problems within the app cannot be solved by the researchers and computer experts are required. So, as researchers, we firstly suggest having enough economical budget to contract with a computer business and have a complete and finalized app before using it for research. Innovative digital tools as pedagogical resources in PE have been previously carried out [39,40,41]. The incorporation of mobile apps into the PE program is also underway [42]. Therefore, the implementation of this app within the educational curricula can enhance the potential benefits.

## 5. Strengths and Limitations

The strengths of this study included the novel AVG app use and its application in educational contexts. In addition, focus groups and qualitative methodology were included by collaborating with experts in this type of analysis. Nevertheless, some limitations must be acknowledged, such as the use of a convenience sample in phase 1, the fact that the AVG was only available for Android software, and the different circumstances and ways of implementing the app between the two phases due to COVID-19.

## 6. Conclusions

The Mystic School app was positively accepted by the adolescents, although the software required some technical improvements (i.e., design and development) for better engagement and enjoyment of the adolescents. Therefore, the app’s shortcomings show that its usability should be improved. After the testing in both phases, it is confirmed that the Mystic School app might be a good game to promote PA by increasing the number of steps. In addition, some technical modifications should be completed regarding the design of the software after experiencing and listening to the participants. Consequently, AVG games, such as Mystic School, are proposed as useful tools in PE lessons. Future works should implement some active methodology activities with the AVG to increase the motivation and adherence of the participants within the PE curriculum.

## Figures and Tables

**Table 1:** Schedule during PE lessons.

PHASE 1 (Play for Two Weeks)		
Title	Description	Duration
Session I. Mystic School presentation	Deep explanation of how the Mystic School app is used.	20 min
Session II. Focus group	Questions about the perception and experience of playing the Mystic School app.	15 min

Personal resources: one researcher; Facilities: classroom; Material resources: computer projector and Internet connection.

**Table 2:** Schedule during PE lessons.

PHASE 2 (Play for One Month)		
Title	Description	Duration
Session I. App presentation	Deep explanation of how the Mystic School app is used.	25 min
Session II. First impressions	General questions on usage and whether they found any difficulties or failures.	10 min
Session III. Knowing failures and progress in the app	Questions about its use, how many steps they recorded, and their current levels in the Mystic School app.	15 min
Session IV. Progress in the app and daily habits	Questions about their general opinions of the Mystic School app.	20 min
Session V. Focus group	Different questions about their perceptions, habits, and implementation regarding the Mystic School app.	10 min

Personal resources: one researcher; Facilities: classroom; Material resources: computer projector and Internet connection.

**Table 3:** Adolescents’ perceptions about the app Mystic School in phase 1.

Content	Phase 1
Design	*“The movement of the avatar is a bit strange because you hit it and it gets stuck with different objects”* *“The movement is a bit uncomfortable. You spend more time trying to make the avatar walk than you do walking in real life”*
Active videogame failures	*“It’s a bit weird, because sometimes I gain a lot of steps when I walk and sometimes, I gain almost no steps at all”* *“The steps magically appear”* *“My screen has locked up and won’t even let me move my avatar”* *“My GPS is working properly, and it does not let me move the avatar”* *“I walked from my house to the school, and I didn’t move from where I was in the video game. I did the same route again and I didn’t move either”*
Difficulties	*“The video game closed by itself, and I had to open it again”* *“In some places the avatar gets stuck and can’t move forward”* *“I advance a level and I don’t really know how I got there”*
Suggestions for improvement	*“Instead of pressing a few seconds to move the avatar, it would be better to move the avatar with the arrows like in other games”* *“There should be a story section and a multiplayer section”* *“We are made for competition. An application to play against others is better than alone”*

**Table 4:** Adolescents’ perceptions about the app Mystic School in phase 2.

Content	Phase 2
Suggestions for improvement	*“I would like to get in the app and that it loads quicker”* *“It would be nice that there is prize that allows you to get into another secret map”* *“Put in the app that enemy players can steal 1000 steps”* *“More achievements, collect more steps and you get a prize because there were levels where you didn’t have to do anything”* *“I would like an offline mode to play when I don’t have Internet connection”*
Playing time	*“My partner wasn’t walking and couldn’t move forward, so I wanted to pass steps to her, but it didn’t work”* *“I put the application in the background and when I opened it again, my avatar appeared in a different place”* *“I have not been able to play because we have a lot of exams and other activities”*
Active video games-positive aspects	*“It’s an original idea”* *“The graphics are good to begin”* *“The concept was well thought out”* *“It’s an entertaining and fun application to promote Physical Activity”*

## Data Availability

Not applicable.

## Supplementary Materials

The following supporting information can be downloaded at: https://www.mdpi.com/article/10.3390/children9121997/s1, File S1. Questions about the application.

## Abbreviations

ACS	Active commuting to/from school
GPS	Global positioning system
MVPA	Moderate-to-vigorous physical activity
PA	Physical activity
PE	Physical education
App	Application
