# Supplementary material for: Testing the Mystic School Mobile Application to Promote Active Commuting to School in Spanish Adolescents: The PACO Study
Source: Children (Basel). 2022 Dec 19;9(12):1997. doi: 10.3390/children9121997 (PMC9776541; doi:10.3390/children9121997)
Supplement: Supplementary file 1 [file children-09-01997-s001.zip › children-2050367-supplementary.pdf]

children MDPI

**Supplementary Materials S1: Questions about different aspects.** The following supporting information can be downloaded at: https://www.mdpi.com/article/10.3390/children9121991/s1, questions about the application.

| Number | Questions |
|---|---|
| 1 | Did you find it easy to pass the level? |
| 2 | Were the objectives set at each level difficult to achieve or too easy? Were they clear? |
| 3 | If you imagine for a moment that the app is yours and you had the power to change, improve or remove something, what would you do? |
| 4 | Would you like the App to have additional functions? Which ones? |
| 5 | What difficulties have you encountered when playing on the App? |
| 6 | At some point in the game you didn't know what to do or felt lost? |
| 7 | What did you like least about the app, and what did you like most? |
| 8 | Would you like the App to allow you to customise the design and appearance of your avatar? In other words, what should it have? |
| 9 | What did you think of the idea of moving around the game through a maze, would you change anything, and can you think of another idea that would be more fun and dynamic? |
| 10 | When you click on the avatar, what information do you think would be relevant to add in that section in addition to the steps? |
| 11 | Is the idea of playing in groups of maximum 3 people useful? Is the app focused on role-playing interesting or would there be another way to make it more fun? |
| 12 | Would you be interested in the App having the function of sharing your achievements and data on the network, would it be useful, in which ones? |
| 13 | Would you like to be able to talk to your group mates in-game via chat? Do you have another idea? |
| 14 | How did you feel when you were playing? What feelings did you have? |
| 15 | What do you think of the App and would you play again? |
| 16 | Would you recommend it to a friend? |
| 17 | Have you noticed any different behaviour than usual while playing the App? What is it? |
| 18 | Do you think this change is positive, negative or indifferent to your life and why? |
| 19 | Have you ever got tired of walking to get around or to pass different levels? |

20 What do you think of the design, we want to know if you feel identified with this model-profile or what things you would change or add to the avatar. If the appearance of the game adapts to the age that is intended (14-15 years old).
